# Deep learning and transfer learning for brain tumor detection and classification

**DOI:** 10.1093/biomethods/bpae080

**Published:** 2024-11-19

**Authors:** Faris Rustom, Ezekiel Moroze, Pedram Parva, Haluk Ogmen, Arash Yazdanbakhsh

**Affiliations:** Computational Neuroscience and Vision Lab, Neuroscience Program, Boston University, Boston, MA, 02215, USA; Computational Neuroscience and Vision Lab, Neuroscience Program, Boston University, Boston, MA, 02215, USA; Department of Radiology, VA Boston Healthcare System, Boston, MA, 02132, USA; Boston University Chobanian & Avedisian School of Medicine, Boston, MA, 02118, USA; Harvard Medical School, Boston, MA, 02115, USA; Department of Electrical & Computer Engineering, Laboratory of Perceptual & Cognitive Dynamics, University of Denver, Denver, CO, 80208, United States; Department of Psychological and Brain Sciences, Computational Neuroscience and Vision Lab, Center for Systems Neuroscience, and Program for Neuroscience, Boston University, Boston, MA, 02215, United States

**Keywords:** convolutional neural networks, brain tumor, MRI T1-weighted image, MRI T2-weighted image, deep dream image, image saliency

## Abstract

Convolutional neural networks (CNNs) are powerful tools that can be trained on image classification tasks and share many structural and functional similarities with biological visual systems and mechanisms of learning. In addition to serving as a model of biological systems, CNNs possess the convenient feature of transfer learning where a network trained on one task may be repurposed for training on another, potentially unrelated, task. In this retrospective study of public domain MRI data, we investigate the ability of neural network models to be trained on brain cancer imaging data while introducing a unique camouflage animal detection transfer learning step as a means of enhancing the networks’ tumor detection ability. Training on glioma and normal brain MRI data, post-contrast T1-weighted and T2-weighted, we demonstrate the potential success of this training strategy for improving neural network classification accuracy. Qualitative metrics such as feature space and DeepDreamImage analysis of the internal states of trained models were also employed, which showed improved generalization ability by the models following camouflage animal transfer learning. Image saliency maps further this investigation by allowing us to visualize the most important image regions from a network’s perspective while learning. Such methods demonstrate that the networks not only ‘look’ at the tumor itself when deciding, but also at the impact on the surrounding tissue in terms of compressions and midline shifts. These results suggest an approach to brain tumor MRIs that is comparable to that of trained radiologists while also exhibiting a high sensitivity to subtle structural changes resulting from the presence of a tumor.

## Introduction

### Convolutional neural networks and transfer learning

Sustained and recent progress in Artificial Intelligence (AI) within last decades has been reaching out to many subfields in medicine including radiology and imaging-based inferences [1–4]. Convolutional neural networks (CNNs) are powerful tools that are iteratively trained on large image datasets for object recognition and classification tasks. CNNs learn to extract salient features pertaining to object classes and associate these features with specific category labels [5]. In doing so, a trained CNN learns to detect these same features in previously unseen testing images, and thus classifies them under the appropriate training label accordingly. CNNs also have the capacity for “transfer learning,” a training paradigm where a model trained on one task can be repurposed for a new, related task using a different dataset while still using the trained weights from the original task [6–8]. This method typically confers an advantage when the original weights prove beneficial when training on the task at hand. Image-based tumor classification involves a difficult pattern recognition problem and can therefore benefit from a transfer learning approach.

### Transfer learning from camouflage animal detection task

Although camouflage animal detection and brain tumor classification tasks involve different images, there might be a parallel between an animal hiding through natural camouflage and a bundle of cancerous cells blending in with the surrounding healthy tissue. The learned process of generalization—the grouping of different appearances under the same object identity—is essential to a network’s ability to detect camouflaged objects and is why such training could prove advantageous for a tumor detection task [9]. A trained CNN’s ability to generalize would be inherited through transfer learning from camouflage animal detection to improve its performance on tumor detection and classification [10].

### Explainable artificial intelligence (XAI) tools

Tracking the performance of a CNN on a tumor detection and classification task across multiple trials is most directly done using an accuracy metric. The testing accuracy of a network is determined by dividing the number of correct classifications of the images by the total number of instances of that category in the testing set that was previously withheld from the network during training. This provides a convenient metric for comparing performances before and after applying camouflage animal transfer learning. Testing accuracy, however, does not describe the changes in the internal state of the network in response to training. This results in the classic artificial intelligence “black box” problem, where the process of the model is unclear, and it is difficult to interpret how a certain result was achieved. To combat this, we uniquely use a combination of three explainable AI (XAI) analysis techniques in conjunction to accuracy metrics: feature spaces, DeepDreamImage, and image saliency mapping. These methods allow us to examine both the imprint of brain tumor training on the models and the effect of the proposed novel transfer learning regimen.

Feature spaces present the CNN’s distribution of every test image relative to one another, providing valuable insight into the network’s organization of data following training. In other words, feature spaces provide a direct demonstration of the internal representation of the data by the network in response to training [11]. By extension, this also demonstrates a network’s generalization ability by showing how different instances of the same object identity are grouped relative to each other. The relationship between individual data points in such a space can be analyzed through the lens of the Universal Law of Generalization, which states that the perceived likeliness of two images increases as the distance between them in a feature space decreases [12].

DeepDreamImage (DDI) is a feature visualization technique that offers an alternative method of visualizing the internal state of a trained CNN. This algorithm recognizes and enhances certain visual patterns perceived by the network in the input data during training, generating a visual ‘prototype’ or ‘feature print’ for each object category. This prototype is essentially a direct visualization of the internal representation of each category by the network. Image saliency maps (or sensitivity maps), on the other hand, demonstrate which features are most salient in each image. This technique generates a heat map over an image that highlights which areas of the input image are most crucial for classification of the image, providing insight into how a network decides classification. The application of these XAI methods is further described in methods.

### Comparison to relevant works

Cutting-edge deep learning models are capable of accurately classifying brain tumors at rates approaching perfection, with several models such as Haque et al. (2024) and Rehman et al. (2020) achieving testing accuracies higher than 98% [13, 14]. Except for Haque et al., XAI application remains limited on most of the highest performance models. In some cases, this is a limitation of model architecture, such as the exceptional 97.77% accuracy ensemble model proposed by Alsubai et al. (2022) [15]. Ensemble models cannot easily apply methods like GradCAM without added steps of superposition and averaging. Regardless, it is good that XAI becomes a priority for models going forward. Table 1 provides comparison of the proposed model with other brain tumor classification models as well as the XAI methods they utilize [13–20].

**Table 1.** Comparison of the proposed model to other brain tumor classification models, including explainability methods.

Reference	Model type	Performance	XAI methods	Notable aspects
Alsubai et al. 2022 [15]	Ensemble neural network for 3-class tumor classification	97.77% accuracy from 10-fold CV (cross validation).	None	Ensemble approach reduces model complexity and increases accuracy with imbalanced datasets
Musthafa M et al. 2024 [16]	Transfer-trained Resnet50 for binary classification of yes or no tumor.	98.52% accuracy.	GradCAM	Epoch-wise GradCAMs provide insight into learning, the model performs strongly but only performs binary classification.
Khan et al. 2022 [23]	Twenty-three-layer CNN combined with pre-trained VGG16	97.8% accuracy, 96.5% precision, 96.4% recall	None	High accuracy across all classes of tumor, architecture is designed to resist overfitting.
Haque et al. 2024 [14]	VGG-19 network filter into Inverted Pyramid Pooling Module to create a U-Net-like architecture.	Very high scores of 99.3% accuracy, 99.2% precision and recall	LIME (local interpretable model-agnostic explanations), feature clustering analysis via t-Distributed Stochastic Neighbor Embedding (t-SNE)	Contains metrics for various models and training procedures (no pretrained weights, no augmentation), ablation table of hyperparameter search for comprehensive documentation of model progression to high accuracy.
Ahmed et al. 2023 [17]	EfficientNet-b0	99.84% F1-score	Shapley Additive Explanations (SHAP)	SHAP analysis coarse resolution, a preprocessing/augmentation regimen well designed to prevent overfit.
AlTahhan et al. 2023 [18]	Best model was the hybrid AlexNet feature extractor with KNN classifier performed best.	97% accuracy, while non-hybrid transfer-trained GoogleNet achieved accuracy of 85%.	None	Tested a variety of CNN architectures to compare with high accuracy hybrid models.
Geetha et al. 2024 [19]	SegNet (U-Net for segmentation) tuned using sine-cosine Archimedes optimization, then fed to Densenet using the same	93% accuracy, 92.3% recall, 92% precision.	None	Proposed tuning algorithm led to increases in accuracy while decreasing computational complexity.
Rehman et al. 2020 [13]	All networks transfer-trained and seed-trained: GoogLeNet, AlexNet, VGG16.	Transfer-trained VGG16 achieved 98.69% accuracy. Transfer-trained AlexNet achieved accuracy of 97.39%	None	Image sharpening and augmentation through rotation and flipping. Compares fine-tuned transfer-trained models with SVMs applied after various feature extractors.
Yan et al. 2023 [20]	VGG-like network with RepOptimizer for classification.	Five-fold CV: 95.46% accuracy, 94.66% precision, 98.32% specificity, 87.11% sensitivity.	GradCAM++, segmentation network.	Classified high-grade gliomas versus low-grade gliomas and included multiple MRI sequences in the dataset. Compared GradCAM results to segmentation network output.
Proposed Model (this work)	AlexNet pretrained on camouflage animal dataset.	T2 transfer-trained network accuracy was 92.20%, T1 transfer-trained network was 87.50%.	GradCAM, feature space analysis, DeepDreamImage	Split T2- and T1-weighted MRIs into separate datasets, compared results of camo pretrain network to basic ImageNet pretrained network. Variety of XAI visualizations.

## Methods

### Building datasets for neural network training

CNNs were trained on a brain tumor classification task using post-contrast T1-weighted and T2-weighted astrocytoma, oligoastrocytoma, oligodendroglioma, and normal (non-cancerous) MRIs obtained from two sources. The majority of our glioma MRIs were obtained from public online repositories such as Kaggle and the Cancer Imaging Archive (TCIA) [21]. For normal MRIs, we used deidentified brain MRIs that was provided to us by co-author Dr Pedram Parva from the Boston VA Healthcare System approved by the Department of Veterans Affairs, VA Boston Healthcare System R&D Committee (Protocol number: [1628677-1]). This source accounted for all normal MRIs, and supplemented our existing glioma dataset. All sources used gathered data under HIPAA compliance, and a CIRB exemption was authorized for the Boston VA Healthcare System data since all provided data was anonymized and deidentified. Age and sex of subjects was not available in public datasets used here and are thus not included in the study. No major preprocessing or spatial normalization was performed on the data to avoid potentially altering the expression of pathologies and introducing error in both the tumoral and peritumoral space [22]. Excessive preprocessing may also distort results from XAI techniques used. Hence, manual image preprocessing in this study only consisted of cropping axial slices from MRI volumes and resizing images as needed to match network input requirements. The resulting dataset consisted of 264 viable axial glioma MRIs: 73 astrocytomas, 44 oligodendrogliomas, 27 mixed tumors formerly described as oligoastrocytomas, and 120 normal.

The camouflage animal datasets used here were imported from a previous project in which neural networks were trained for the detection of camouflage animals [10]. It consisted of almost 3,000 images of clear and camouflaged animals divided into 15 categories.

### Neural network training parameters

AlexNet is a CNN consisting of 8 learnable layers, imported via MATLAB version 2024a. AlexNet is pretrained on over a million images in up to 1000 object categories, and was used as the basis of the trained networks described below. Technically, all models described here are transfer trained since we are using pretrained AlexNet weights at baseline. However, in this paper we are using the term transfer learning to describe models that have had an additional sequence of transfer learning beyond its initial baseline.

**Figure 1.**
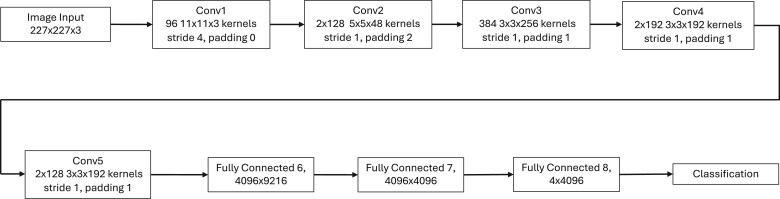
Flow chart of AlexNet architecture, from image input (top left) to classification outcome (bottom right)

AlexNet was deliberately chosen as a well-suited model for the task at hand given the data available instead of more complex, state of the art models employed in similar studies. The less complex architecture of AlexNet, shown in Figure 1, leaves it less prone to overfitting when trained on smaller datasets, as is the case in this study where large-scale MRI datasets are not readily available. Overfitting occurs when a network is able to achieve better results on training than testing, indicating it has memorized features of the dataset that were not generalizable to the overall category. This phenomenon has been documented by other studies using similar, larger models to perform tumor classification [23]. It was key to avoid overfitting given our dataset’s limited size while only using one network to preserve capability of XAI visualization tools.

All images were resized to 227x227 pixels to fit the AlexNet input requirements and were randomly split into training (70%) and testing (30%) sets patient-wise, such that images from the same patient were not cross-contaminated between sets to avoid overfitting. The T1 post-contrast sets were split more evenly to account for the smaller dataset size by having a reasonable amount of testing images. Training was performed on the same machine with the following parameters: an initial learn rate of 0.001, a learn rate drop factor of 0.1, a piecewise learn rate schedule, a validation frequency of every 6 epochs, with the validation set shuffled every epoch, for a maximum of 24 training epochs using a stochastic gradient descent with momentum learning algorithm and the cross-entropy loss function. The loss function is shown in equation 1, where *N* is the number of observations in a minibatch, *J* is the number of classes, *T* is the probability an observation belongs to a given class, and *Y* is the true class vector [24].

(1) $$loss=-\frac{1}{N}\sum_{i}^{N}\sum_{j}^{J}T_{ij}lnY_{ij}$$

Full modeling and data analysis code can be accessed with the following link: https://github.com/frustom/Brain-Tumor-Classification (ID: 6fd7c06)

### Training neural networks on datasets

Two neural networks were trained in this project on the detection and classification of post-contrast T1 and T2 MRIs: T1Net and T2Net, respectively. The dataset included astrocytomas, oligoastrocytomas, and oligodendrogliomas as the tumor categories, and normal brain MRIs as a control. Although we recognize the outdated oligoastrocytoma label [25], we included the images since they were readily available in the datasets used and thus serve as an additional challenge for the neural network to classify appropriately with respect to the other glioma classes.

**Figure 2.**
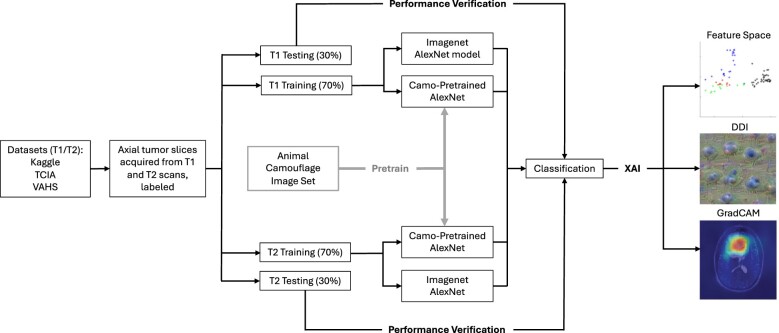
Flow chart showing pipeline of study methods, starting from dataset acquisition (left), through network training, and finally XAI interpretation of the network’s internal state. Important regions of the MRI are shown by GradCAM, while feature visualization and representation are shown by DDI and feature space

The previously mentioned camouflage animal detection networks were used as seeds for the tumor classification networks ExpT1Net and ExpT2Net, which both used the ExpCamoNet layer weights as a starting point for training. The entire pipeline is visualized in Figure 2.

### Dimensionality reduction and feature spaces

**Figure 3.**
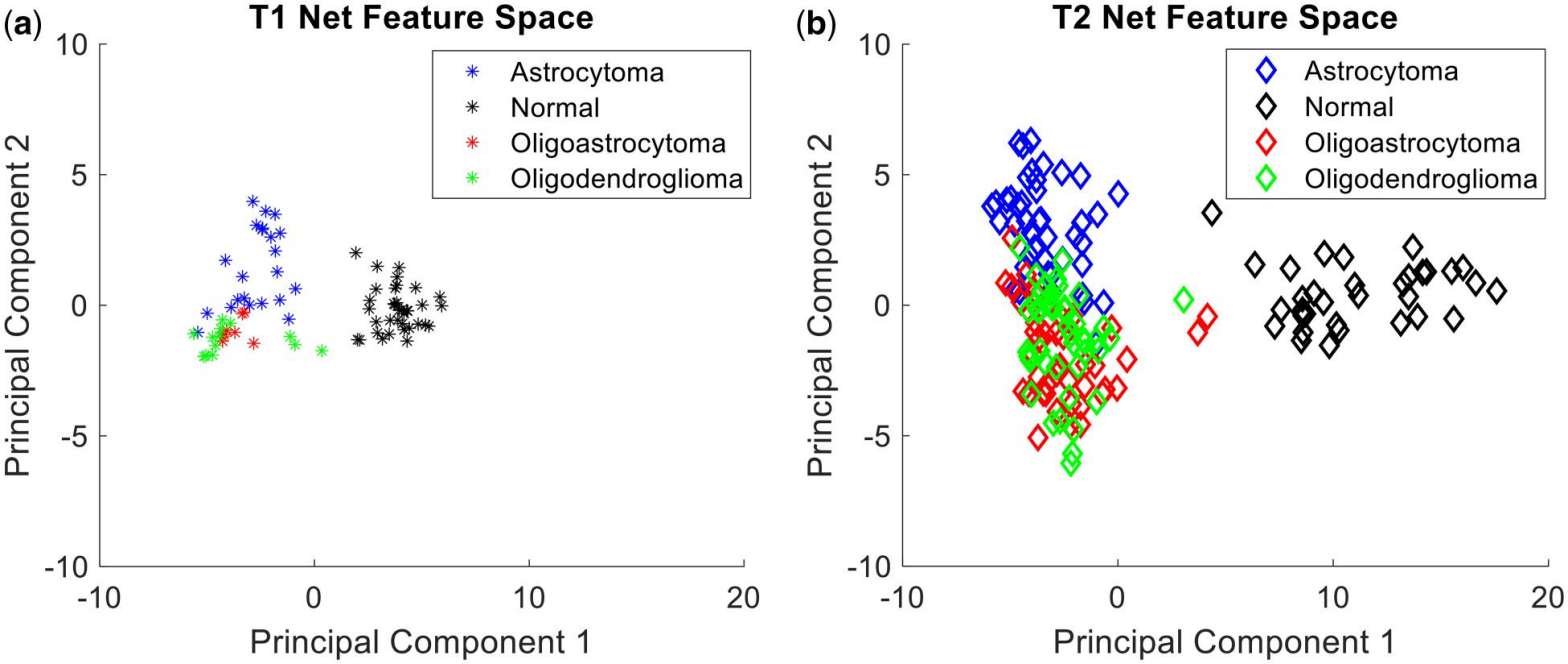
Feature spaces for T1Net (**a**) and T2Net (**b**) across first two principal components

**Figure 4.**
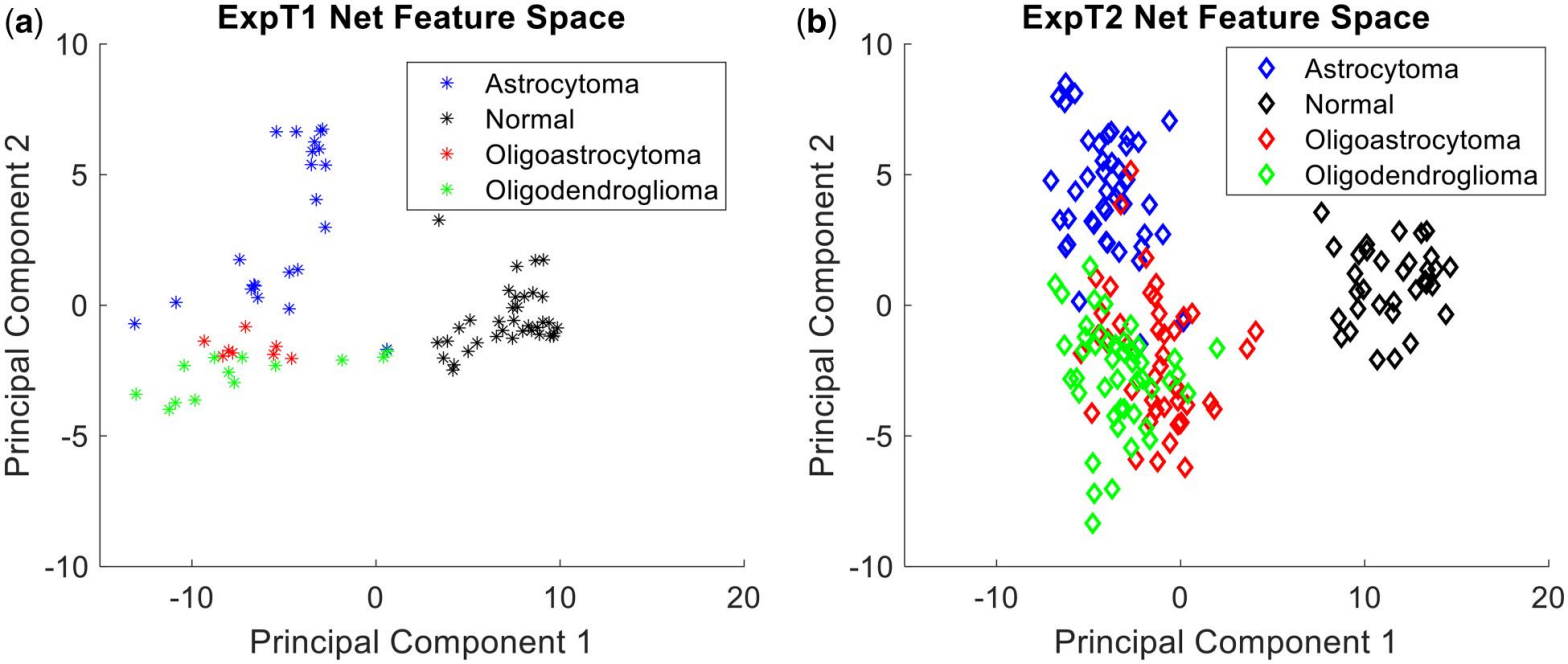
Feature spaces for ExpT1Net (**a**) and ExpT2Net (**b**) across first two principal components

To map the feature spaces (examples in Figures 3-4), the vector of activation values of each category node was extracted from the Fully Connected (FC) layer of a CNN. This vector contains the activation values of every testing image across all category nodes. Principal Component Analysis (PCA) was used as a dimensionality-reduction method to simplify the larger activation matrices to three key dimensions that are representative of the overall activation values. The first three Principal Components were then plotted to model the networks’ feature space in three dimensions. In these feature spaces, each point represents the activation of a single testing image on the trained FC layer nodes and its position within the feature space, as part of a color-coded region, is a visualization of the networks’ internal representation of the data. This XAI technique thus demonstrates how the networks group tumor categories relative to each other, based on similarities and differences in their perceived visual characteristics.

### DeepDreamImage

DeepDreamImage (DDI) is a feature visualization technique that offers an alternative method of visualizing the internal state of a trained CNN [26]. This gradient ascent (as opposed to gradient descent) algorithm passes an image with pixel values randomly sampled from a normal distribution through the network and calculates the derivative of the image with respect to the activation values of the chosen layer [27]. The algorithm seeks to maximize the loss, which is the sum of the activations in that layer, for the image to increase the activation of that layer. The gradients of this new ‘excited’ image are calculated with respect to the original image, and then added to the original image. The resulting image of this XAI method is an interpretation of basic features the network associates with a respective class, which helps clarify the rationale behind a classification decision. By amplifying the distinctive patterns of each tumor class, we can see a network’s visual ‘prototype’, or ‘feature print’ for a given tumor type.

### GradCAM saliency maps

To determine which features of a given image were most important for the neural networks decision making after training, image saliency maps were generated to visualize feature importance. The GradCAM (gC) MATLAB function was used to generate heat maps overlaying select images of each training category representing the intensity of the network’s sensitivity to a given area [28]. The gC function pools classification score gradients with respect to convolutional features to find the neuron importance weights, which are used to determine which features are most crucial for classification [29]. To compute the gC map for a convolutional layer, A, with k channels, where i,j indexes the pixels, in a CNN with output y with class score c, the neuron importance weight is

(2) $$\alpha_{k}^{c}=\frac{1}{N}\sum_{i}\sum_{j}\frac{\partial y^{c}}{\partial A_{i,j}^{k}}\;,$$

where N is the total number of pixels in the channel. To generate the gC map, a weighted combination of the channels is then calculated with an applied ReLU

(3) $$M=\mathrm{ReLU}\left(\sum_{k}\alpha_{k}^{c}A^{k}\right).$$

We applied this function on activation values in both the softmax and fully connected layers to generate image saliency maps for both cancerous and normal subjects on all trained neural networks. The resulting heat maps are a useful XAI tool for determining which areas of the input MRI images are most valuable to the network when classifying tumors. The deeper red on the heat map, the more salient (important to classification outcome) the area is.

## Results

### Model performance before and after camouflage animal transfer learning

Both T1Net and T2Net showed a modest performance on the glioma classification task. T1Net had an average accuracy of 85.99%, with T2Net trailing behind at 83.85%. Table 2 outlines the class-specific and overall model accuracies and statistics of each trained network. T1Net and T2Net both had near perfect accuracies on normal brain images, with only 1-2 false negatives between both networks, demonstrating a strong ability to differentiate between cancerous and normal brains. The networks struggled more with glioma subtype classification, with each network showing contrasting performance on glioma categories. T1Net’s best glioma category was astrocytoma (95.46%) and its worst was oligoastrocytoma (12.50%), whereas T2Net’s best category was oligoastrocytoma (93.33%) and its worst was astrocytoma (74.42%).

**Table 2.** Mean (± CI) model performance, class-specific accuracies, and sample training/testing data splits across T1- and T2-weighted images, for both seed- and transfer-trained networks. Data splits are identical for transfer trained networks, and hence not listed.

Network	Trained On	Tested On	Accuracy (%)
T1Net	T1 MRIs	T1 MRIs	85.99
	Astrocytoma (44 training, 29 testing)		95.46
	Oligoastrocytoma (15 training, 12 testing)		12.50
	Oligodendroglioma (31 training, 13 testing)		61.54
	Normal (86 training, 34 testing)		97.30
T2Net	T2 MRIs	T2 MRIs	83.85
	Astrocytoma (108 training, 37 testing)		74.42
	Oligoastrocytoma (105 training, 45 testing)		93.33
	Oligodendroglioma (111 training, 43 testing)		82.61
	Normal (67 training, 45 testing)		94.44
ExpT1Net	Camo animals → T1 MRIs	T1 MRIs	87.50 ± 2.28
			p = 0.3153
		Astrocytoma	95.46
		Oligoastrocytoma	100
		Oligodendroglioma	53.85
		Normal	92.31
ExpT2Net	Camo animals → T2 MRIs	T2 MRIs	92.20 ± 3.68
			p = 0.0035
		Astrocytoma	93.02
		Oligoastrocytoma	88.89
		Oligodendroglioma	97.83
		Normal	100

The previously trained camouflage animal detection model, ExpCamoNet [10], was used as the seed network for training two transfer trained networks, ExpT1Net and ExpT2Net. Both transfer trained networks achieved a higher mean accuracy than their non-transfer trained counterparts (Table 2). The accuracy increase was only significant in the case of ExpT2Net (83.85%–92.20%, *P* = .0035, CI = 3.68), whereas ExpT1Net experienced a much smaller increase in average accuracy (85.99%–87.5%, *P* = .3153, CI = 2.28). The accuracy of the normal category remained near-perfect in the case of ExpT1Net, while ExpT2Net rectified the prior false negatives to achieve perfect accuracy on normal images. ExpT1Net’s largest improvement was in the oligoastrocytoma category, jumping from 12.50% to 100%, with all other categories remaining relatively unchanged. ExpT2Net improved in classification of each category, most notably in its formerly worst astrocytoma category (74.42%–93.02%, also compare Figures 3b and 4b).

### Feature space transformations in response to transfer learning

The feature spaces of both T1Net and T2Net reveal a distribution of the data in each category relative to another by the networks in a two-dimensional space (Figure 3). We observe a similar pattern of data distribution across both T1Net and T2Net, in which there is a distinct separation between the normal category region and the three glioma category regions and the normal category region on the first principal component. This is consistent with the near-perfect normal image accuracy in both networks mentioned above as it translates to a clear decision boundary between these categories. The three glioma category regions, on the other hand, are blended in both the T1Net and T2Net feature spaces across both the first and second principal components. This represents a more muddled decision boundary between the categories by the networks, again consistent with the lower accuracies for these categories. Despite the glioma categories being mixed together, it is apparent upon closer inspection that the oligoastrocytoma category region mostly occupies the space between the astrocytoma and oligodendroglioma regions in both networks.

When comparing the feature spaces of ExpT1Net and ExpT2Net after transfer learning to their original versions, we notice changes in data distribution (Figure 4). As expected, the normal category region still occupies an independent region separate from the glioma categories as before, albeit with some noticeable increase in separation on the first principal component between normal and glioma regions indicative of the expansion of feature space in ExpT1Net. The more obvious transformation is within the glioma categories themselves in the ExpT2Net feature space (Figure 4b), which had the more pronounced quantitative improvement in accuracy. This translates to increased separation of the astrocytoma category region to the point of now showing a much more defined decision boundary, especially when compared to the T2Net feature space (Figure 3b) in which astrocytoma was the worst performing category. This is also true to a lesser extent for the oligoastrocytoma and oligodendroglioma category regions which remained mixed together, but now with more distinct separation between them. The oligoastrocytoma category remains in between the astrocytoma and oligodendroglioma regions as before. These observations hold true for the ExpT1Net feature space, but to a lesser extent since transfer learning was not as beneficial to this network despite the expansion of feature space in ExpT1Net compared to T1Net.

### DeepDreamImage analysis of trained networks

**Figure 5.**
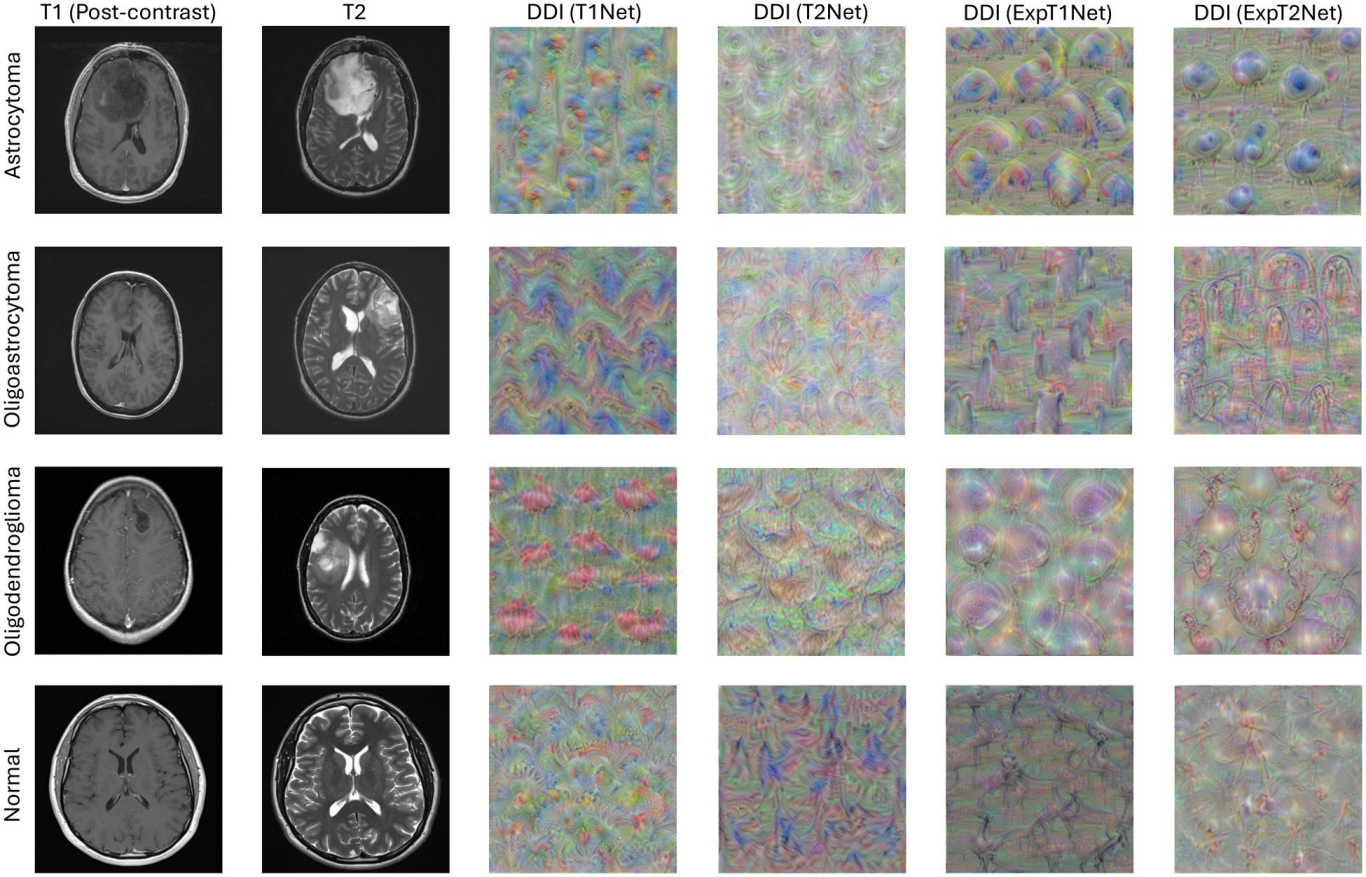
DeepDreamImage (DDI) images of each category of tumor (rows) generated by each trained network (columns 3–6). Sample post-contrast T1-weighted (column 1) and T2-weighted (column 2) MRIs are shown for visual comparison of tumor characteristics (*feature prints)* to those generated through DDI. Feature prints appear to have more distinct forms and shapes for all tumors relative to control (rows 1–3), with transfer learning further enhancing and sharpening the shape of feature prints (columns 5–6)

DeepDreamImage (DDI) images of each data type for the four trained networks were generated to provide an additional qualitative assessment of the networks’ internal representation of glioma and normal brain MRIs. Figure 5 shows a side-by-side comparison of each DDI produced per network and category. The seed networks, T1Net and T2Net, appear to produce certain shapes corresponding to the tumor categories they were trained on. For example, the astrocytoma DDI shows a repeating nodal shape pattern throughout the image, whereas the oligodendroglioma DDI shape is more bulbous. The oligoastrocytoma DDI shape falls into an intermediate of the two other glioma categories, appearing to have both a central node and an external bulb shape.

After transfer learning, the ExpT1Net and ExpT2Net DDI-generated images have similar patterns that are more pronounced. Clearer and more defined versions of the shapes described in T1Net and T2Net DDI emerge, while retaining their original node/bulb characteristics. This again represents how transfer learning enables the networks to produce more distinct internal representations of the data. For normal image DDIs, on the other hand, they seem to lack any clear shapes like those observed in glioma DDIs. This suggests that the networks associate a lack of distinct features with the normal category classification label, while associating pronounced nodal and bulbous shapes with the various glioma categories.

### GradCAM analysis

**Figure 6.**
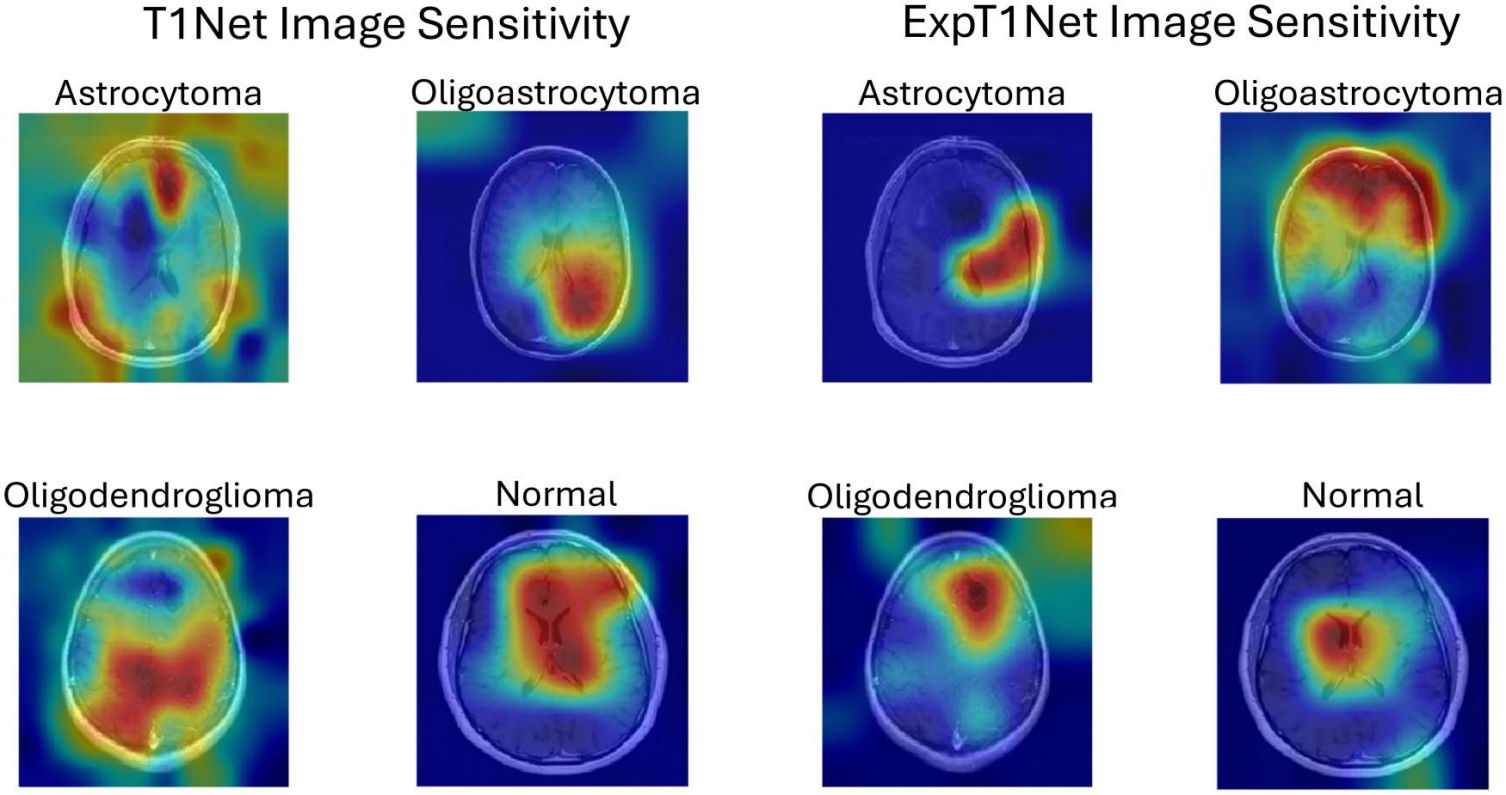
Image sensitivity maps for T1Net and ExpT1Net showing transfer learning effect. Sensitivity maps were generated using GradCAM, on both softmax and fully connected layers. The underlying sample images are the same as those from Figure 5. Warmer hues (more red) indicate higher saliency, or more importance to classification outcome

**Figure 7.**
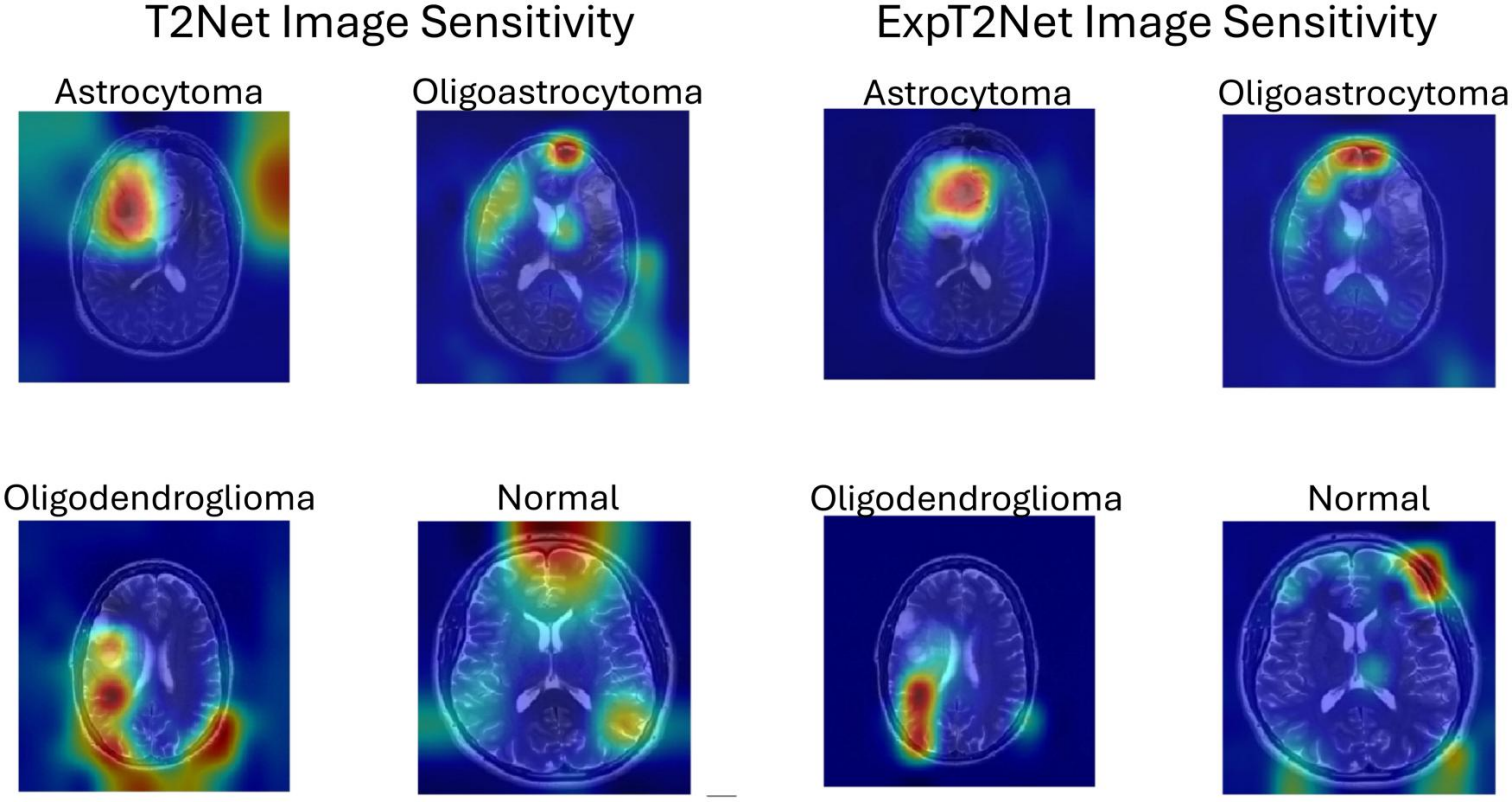
Image sensitivity maps for T2Net and ExpT2Net showing transfer learning effect. Sensitivity maps were generated using GradCAM, on both softmax and fully connected layers. The underlying sample images are the same as those from Figure 5. Warmer hues (more red) indicate higher saliency, or more importance to classification outcome

GradCAM saliency maps provide another layer of analysis for identifying salient features used by a network to make a classification decision. Figures 6 & 7 show the side-by-side comparison of the saliency heatmaps across all image categories and networks. The saliency maps for T1Net and T2Net show that these networks typically identify the tumor itself as the most salient feature of the image, as one would expect. In the case of T1Net especially, the areas of highest saliency are broad and cover a sizable portion of the brain’s surface (Figure 6). In certain instances (T1 oligoastrocytoma, T1 oligodendroglioma, T2 oligoastrocytoma, and T2 oligodendroglioma) the networks appear to also focus on the tissue adjacent to the tumor but not directly on the tumor itself.

After transfer learning, both ExpT1Net and ExpT2Net’s saliency maps show a more precise and specific focus on certain features compared to the broader sensitivity in the seed networks. Again, this includes sensitivity to either the tumor itself and/or the tissue around it as it becomes distorted by the presence of the tumor. When looking at the image saliency maps of the normal images, for both seed and transfer trained networks, the most salient areas are either in the middle of the brain (in the case of T1Net and ExpT1Net, Figure 6) or at the borders of the skull (T2Net and ExpT2Net, Figure 7).

## Discussion

This investigation is the first of its kind to apply camouflage animal transfer learning to deep neural network training on a tumor detection and classification task. Our results demonstrate that this approach to deep neural network training is promising, specifically when using T2-weighted MRI data, as this model showed the greatest improvement in testing accuracy. While this best performing proposed model is about 6% less accurate than the best shown in Table 1, we successfully demonstrate the quantitative improvement brought on by this training paradigm. Our use of feature spaces, DeepDreamImage, and GradCAM saliency maps effectively communicates the internal states of our models and how camouflage animal transfer learning influences their decision-making process. These XAI methods allow us to insightfully visualize what is happening as the models train on brain cancer imaging data and what characteristics it associates with different tumor classes, providing a dimension of analysis beyond accuracy metrics.

The feature spaces of the trained networks showed an interesting organization of the glioma data. In each case, the oligoastrocytoma category region occupied the space in between the astrocytoma and oligodendroglioma regions. Even though oligoastrocytomas are no longer recognized as a glioma, rather now considered mixed cell tumors, we used the available oligoastrocytoma data in this study to evaluate the networks’ performance with its inclusion. We found that the networks intuitively recognized the mixed cell appearance of oligoastrocytomas as an intermediate between astrocytomas and oligoastrocytomas, and thus organized them as such relative to the two other glioma categories. In other words, the networks’ internal representation of oligoastrocytomas is somehow consistent with the updated classification of gliomas.

The feature spaces also illustrate an increased generalization ability of the neural networks after transfer learning accompanied with expanded region of each category in feature spaces. As described in the Results section, transfer learning led to an increase in mean accuracy for the networks as well as feature space transformations consistent with this increase. The increased separation between category regions represents a more defined decision boundary by the network, which is consistent with an enhanced generalization ability gained by the networks from camouflage animal transfer learning. Since the process of camouflage animal detection itself is heavily reliant on the ability to generalize the appearance of an animal under different conditions, it is possible that this ability was abstracted by the tumor detection networks during the transfer learning process. ExpT2Net performs better than T2Net (8.35% accuracy increase, *P* = 0.0035) because it gained the ability to generalize the appearance of the tumors under differing conditions and thus detect it more consistently than before. ExpT1Net did not see a significant increase in accuracy over T1Net (1.51% accuracy increase, *P* = 0.3153), but Figure 4 reflects expanded generalizability in the feature space similarly to ExpT2Net.

DeepDreamImage images generated by the transfer trained networks reinforce our idea of increased generalizability for both Exp networks. To better understand how DeepDreamImage reinforces this concept, we must analyze the generated images through the lens of *“feature prints”*. Each independent object identity is imprinted onto a network in a unique way that makes it separate from all other object identities, in the same way that a fingerprint is unique to each individual. Therefore, an astrocytoma’s imprint on a neural network is different from that of a oligodendroglioma, which is different from that of an oligoastrocytoma or a normal brain. With DeepDreamImage, we can visualize these unique imprints in terms of the features that come to be associated with each category (in other words, their feature print). Figure 5 shows the feature print images generated for each image category (rows) by each network (columns 3–6).

In Figure 5 we can see that the basic elements of feature prints are consistent across T1Net/ExpT1Net and T2Net/ExpT2Net, where it was noted previously that astrocytomas have a nodal shape feature print, oligodendrogliomas have a bulbous shape feature print, and oligoastrocytomas have a mixed feature print containing nodal and bulb characteristics. These feature prints appear regardless of if camouflage animal transfer learning was used, but they become more defined and pronounced after transfer learning. This is another indication that transfer learning endows the networks with an enhanced generalization ability to detect these key tumor feature prints across varying brain MRIs and classify them accordingly.

The GradCAM saliency maps exhibit a high-level decision-making process by the networks to detect and classify tumors. In several cases, the networks did not highlight the tumor as the most salient feature. Rather, it focused on the surrounding tissue directly adjacent to the tumor, and even in more distal regions in some cases. This approach of examining the distortion of neighboring tissue caused by the tumor is similar to that of radiologists when studying similar cancerous MRIs [30], which the network determined without prior suggestion. Specifically, the perceived saliency to brain regions directly opposite the tumor itself in some cases suggests an ability of the network to compare symmetrical regions for structural displacements and shifts secondary to tumors, representing a highly sensitive feature of the network. Through training on the glioma data, the network learned to look at the areas affected by the tumor and not just at the tumor itself when making a classification decision.

This study had some limitations. First, the imbalance of the datasets may have affected the networks’ performance on certain categories. The datasets used contained more T2-weighted MRIs than post contrast T1-weighted, which may explain the discrepancy between ExpT1Net and ExpT2Net after transfer learning. Furthermore, the number of images within each category was imbalanced, especially with T1-weighted images. For example, there were more post-contrast T1 astrocytoma MRIs than any other category, which is likely why T1Net performed best on that category relative to the other glioma types. Second, the lack of standardization between images from different dataset sources may have factored into the networks’ performance. The MRIs from TCIA were slightly different in formatting than those obtained from the VA healthcare system, and these differences may have influenced networks’ classification decisions.

In conclusion, CNN models were trained on glioma detection and classification in post-contrast T1-wieghted and T2-weighted brain MRIs. A transfer learning from previously trained camouflage animal detection models was shown to improve performance accuracy on this task, specifically in the case of T2-weighted images (*P* = .0035, CI = 3.68). The highest performing model achieved perfect accuracy on normal brain MRIs with no false negatives or positives, and near perfect accuracy on classifying astrocytomas and oligodendrogliomas. Qualitative XAI methods were used to provide a deeper analysis of the models’ performance in terms of the internal transformations resulting from training, which all independently suggested an improved generalization ability following camouflage animal transfer learning. Specifically, employment of these methods revealed a tumor-specific feature-based approach to glioma classification taken by the models during the decision-making process. Our models have been made publicly available for further investigation and potential clinical application in glioma detection and classification from MRI data for the benefit of the scientific community. Follow up research could utilize the novel camo transfer learning regimen with more complex models with more available data, which could possibly increase generalizability and improve model performance beyond the current cutting edge.
